# Application of metabolomics in drug-induced liver injury: A systematic review

**DOI:** 10.1515/jtim-2026-0056

**Published:** 2026-08-12

**Authors:** Xiaoyan Sun, Xiaotong Li, Xiaona Li, Siyao Jin, Na Wang, Meng He, Lijuan Zhou, Wei Zhang, Xin Xiong, Libo Zhao

**Affiliations:** Department of Pharmacy, Peking University Third Hospital, Beijing, China; Department of Pharmacy, Zhengzhou Central Hospital Affiliated to Zhengzhou University, Zhengzhou, Henan Province, China; School of pharmacy, University of Pittsburgh, Pittsburgh, PA, USA; Therapeutic Drug Monitoring and Clinical Toxicology Center of Peking University, Beijing, China

**Keywords:** drug-induced liver injury, metabolomics, biomarkers, prediction, diagnosis

## Abstract

Drug-induced liver injury (DILI) is one of the most common adverse reactions in clinical settings, and drug hepatotoxicity is also a primary factor contributing to the suspension and withdrawal of drug development. Currently, there are no specific biomarkers available for the early diagnosis and monitoring of DILI in clinical practice. In recent years, metabolomics has emerged as a powerful tool for identifying small molecule biomarkers in DILI, owing to its unique advantages. In this article, we comprehensively reviewed the metabolomics studies related to DILI to investigate the pathophysiological changes in DILI and to assess the potential application of metabolomics in its prediction and diagnosis. Relevant literature published before 30 April 2024 was retrieved from four online databases (PubMed, EMbase, Cochrane Library, and Web of Science). Research data were systematically collected, and the metabolites involved were analyzed. Metabolic pathway analysis related to DILI was conducted using the online software MetaboAnalyst 5.0. A total of 55 studies were included, identifying significantly altered metabolic pathways and valuable metabolic biomarkers. The most frequently reported biomarkers included glycocholic acid, taurochenodeoxycholic acid, taurocholic acid, creatine, glycochenodeoxycholic acid, taurodeoxycholic acid, glycodeoxycholic acid, and *α*-ketoglutaric acid. The disrupted metabolic pathways were mainly related to bile acid metabolism, lipid metabolism, and the tricarboxylic acid cycle, among which bile acid metabolism showing the most prominent alterations. Lipid metabolism-related pathways included fatty acid biosynthesis, glycerophospholipid metabolism, and sphingolipid metabolism. This article provides a systematic review of metabolomics in DILI, deepening our understanding of the metabolic disturbances caused by DILI.

## Introduction

Drug-induced liver injury (DILI), a form of hepatotoxicity, is characterized by hepatic damage attributed to pharmaceuticals, herbal preparations, nutraceuticals, and dietary supplements.^[1]^ As a prevalent clinical presentation of hepatotoxicity,^[2]^ DILI is a leading cause of the termination of drug development pipelines and post-marketing drug recalls. Consequently, it constitutes a significant safety and financial concern for the pharmaceutical sector and healthcare systems, highlighting its relevance to public health.^[3]^ The diagnosis of DILI is inherently complex due to the absence of pathognomonic clinical features and definitive diagnostic assays.^[4]^ Despite the proliferation of hypotheses regarding DILI, a conclusive etiological link between medications, associated risk factors, and the pathophysiological mechanisms of DILI remains to be definitively established.^[5, 6, 7]^ The Roussel Uclaf Causality Assessment Method (RUCAM) remains an important tool for assessing DILI at present, in spite of the challenges of a complex and cumbersome operation process, as well as the high demands on the professional skills of physicians and clinical pharmacists.^[8, 9]^ This diagnostic approach necessitates a multifaceted evaluation that includes heightened clinical suspicion, a comprehensive patient history encompassing risk factors and their chronological progression, and an in-depth hepatological examination. In summary, the intricate nature of pharmaceutical compounds, variability in prescribing practices, incomplete comprehension of population diversity, the pathogenesis of DILI, and the spectrum of clinical phenotypes, coupled with the scarcity of specific diagnostic biomarkers and efficacious therapeutic interventions, render the timely detection, diagnosis confirmation, prognostic assessment, clinical stewardship, and risk mitigation of DILI a formidable challenge.^[10]^ Consequently, the pursuit of novel diagnostic biomarkers facilitated by cutting-edge technologies is of paramount importance.

Metabolomics, an emerging field within systems biology, is recognized as a robust phenotyping technique that provides a direct and holistic assessment of the dynamic biochemical profiles within biological systems. It qualitatively and quantitatively characterizes the metabolic alterations in living organisms, effectively delineates perturbed metabolic pathways within cellular and tissue contexts, and elucidates the interplay between metabolites and physiological or pathological alterations. This approach is instrumental in dissecting the complex human responses to diseases and therapeutic interventions.^[11, 12]^ Within the realm of hepatology, metabolomics has gained traction for its utility in investigating liver pathologies, particularly those induced by xenobiotics. Consequently, the integration of metabolomics into DILI research facilitates the identification of specific metabolites and metabolic pathways associated with DILI. This, in turn, deepens our understanding of the underlying etiology and pathophysiological mechanisms of DILI, ultimately facilitating the development of more effective clinical strategies for its early detection, diagnosis, and therapeutic management.

In this systematic review, we exhaustively screened metabolomics studies related to DILI, extracting and analyzing pertinent data. Our synthesis highlighted significant fluctuations in metabolic biomarkers and pathways associated with DILI, advancing our understanding of its etiology and pathogenesis, and evaluating the prognostic and diagnostic potential of these biomarkers.

## Methods

The systematic review was carried out following the Preferred Reporting Items for Systematic Reviews and Meta-Analyses (PRISMA) guidelines.^[13]^ The protocol for this systematic review was registered on the International Prospective Register of Systematic Reviews (PROSPERO) platform (Registration No: CRD42025637672).

### Literature search

We conducted a systematic search of the metabolomics literature on DILI through 30 April 2024, utilizing four electronic databases: Web of Science, PubMed, EMBase, and the Cochrane Library. The search strategy was designed as follows: “Drug induced liver injury” [All Fields] AND (“metabolome” [MeSH Terms] OR “metabolome” [All Fields] OR “metabolomes” [All Fields] OR “metabolomics” [MeSH Terms] OR “metabolomics” [All Fields] OR “metabolomic” [All Fields]). The screening and assessment for study inclusion were performed independently by two authors, with a third researcher adjudicating any discrepancies.

### Criteria for inclusion and exclusion

Inclusion criteria were as follows: (1) Studies on metabolomics-focused investigations, applying standardized metabolomics platforms (including nuclear magnetic resonance (NMR), gas chromatography-mass spectrometry (GC-MS), liquid chromatography-mass spectrometry (LC-MS), and atomic absorption spectroscopy (AAS), *etc*.), reporting key analytical validation parameters, and including multivariate statistical analysis; (2) Studies have a full text or abstract in English with complete information, including complete methodological descriptions (sample preparation, instrumentation, data processing) and availability of essential outcomes; (3) Studies with positive or negative correlation between DILI and metabolomic markers, including quantitative demonstration of biomarker-DILI correlation, reporting of diagnostic performance metrics (AUC, sensitivity, specificity) and mechanistic interpretation of metabolic pathways.

Exclusion criteria were as follows: (1) Review articles, comprising systematic reviews, meta-analyses, narrative reviews, editorial commentaries, conference abstracts without full data and methodological papers without biological validation; (2) *In vivo* animal models (rodents, primates, *etc*.), *in vitro* cell culture experiments (primary hepatocytes, cell lines), and ex vivo organ perfusion models; (3) Studies assessing drug efficacy, pharmacokinetic studies without metabolomic profiling, investigations of therapeutic interventions on established DILI.

### Data extractions

Upon full-text review and examination of supplementary materials, we systematically extracted the following data: (1) study characteristics comprising principal investigator, publication date and geographical location; (2) basic subject information including medication profiles, clinical laboratory metrics, and subject stratification; (3) research methodologies, diagnostic criteria for DILI/hepatotoxicity, biological sample types, and metabolomic platforms; (4) metabolic markers and metabolic pathways exhibited significant changes. Furthermore, we scrutinized for and addressed any potential duplication of content across studies authored or co-authored by the same individuals.

### Statistical analysis

Study characteristics were visualized using GraphPad software (Prism 8). Pathway enrichment and pathway analysis of significantly changed biomarkers was done by MetaboAnalyst 5.0 online software (https://www.metaboanalyst.ca).

## Results

### Characteristics of the studies

**Figure 1:**
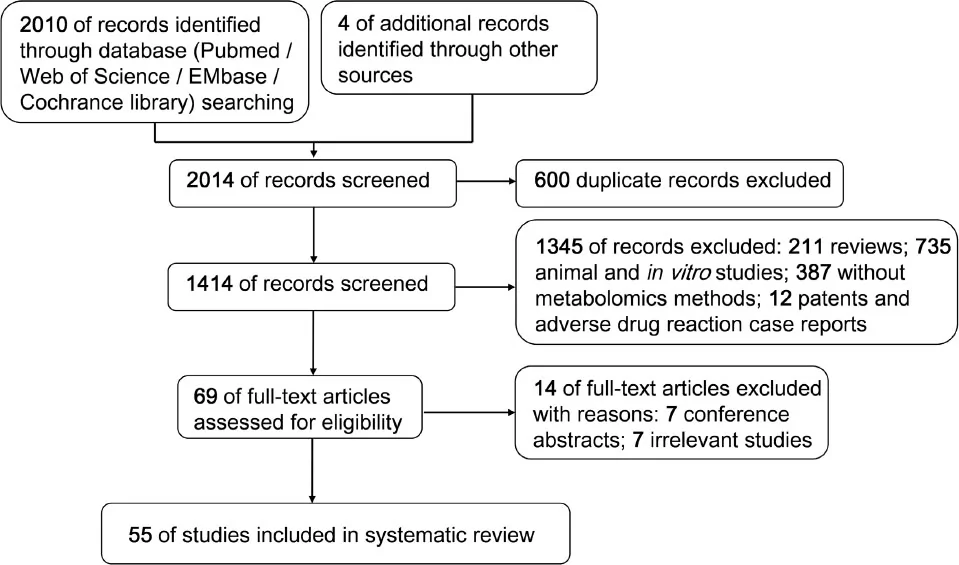
Flowchart for literature search and literature selection for metabolomic markers of DILI. DILI: drug-induced liver injury.

This systematic review was conducted through an initial comprehensive literature search of multiple databases, resulting in the identification of relevant studies. Following the application of rigorous, predefined inclusion and exclusion criteria, a final cohort of 55 studies was selected for in-depth analysis.^[14, 15, 16, 17, 18, 19, 20, 21, 22, 23, 24, 25, 26, 27, 28, 29, 30, 31, 32, 33, 34, 35, 36, 37, 38, 39, 40, 41, 42, 43, 44, 45, 46, 47, 48, 49, 50, 51, 52, 53, 54, 55, 56, 57, 58, 59, 60, 61, 62, 63, 64, 65, 66, 67, 68]^ The process of literature screening and study inclusion is depicted in Figure 1. The 55 included studies are detailed across Table S1 to Table S5: Table S1 enumerates 17 investigations on acetaminophen-induced hepatotoxicity; Table S2 summarizes studies on hepatotoxicity related to herbal medicines (*Polygonum multiflorum* [PM] and pyrrolizidine alkaloids); Table S3 focuses on liver injuries caused by exogenous chemical toxins, including arsenic trioxide, polyvinyl chloride, and cadmium; Table S4 presents 11 studies on drug-induced liver injury caused by specific single agents or drug classes, including voriconazole, ethanol, amoxicillin, valproate, simethicone, methimazole, and anti-tuberculosis medications; Table S5 aggregates 19 studies with complex medication profiles, predominantly examining multi-drug induced hepatotoxicity.

**Figure 2:**
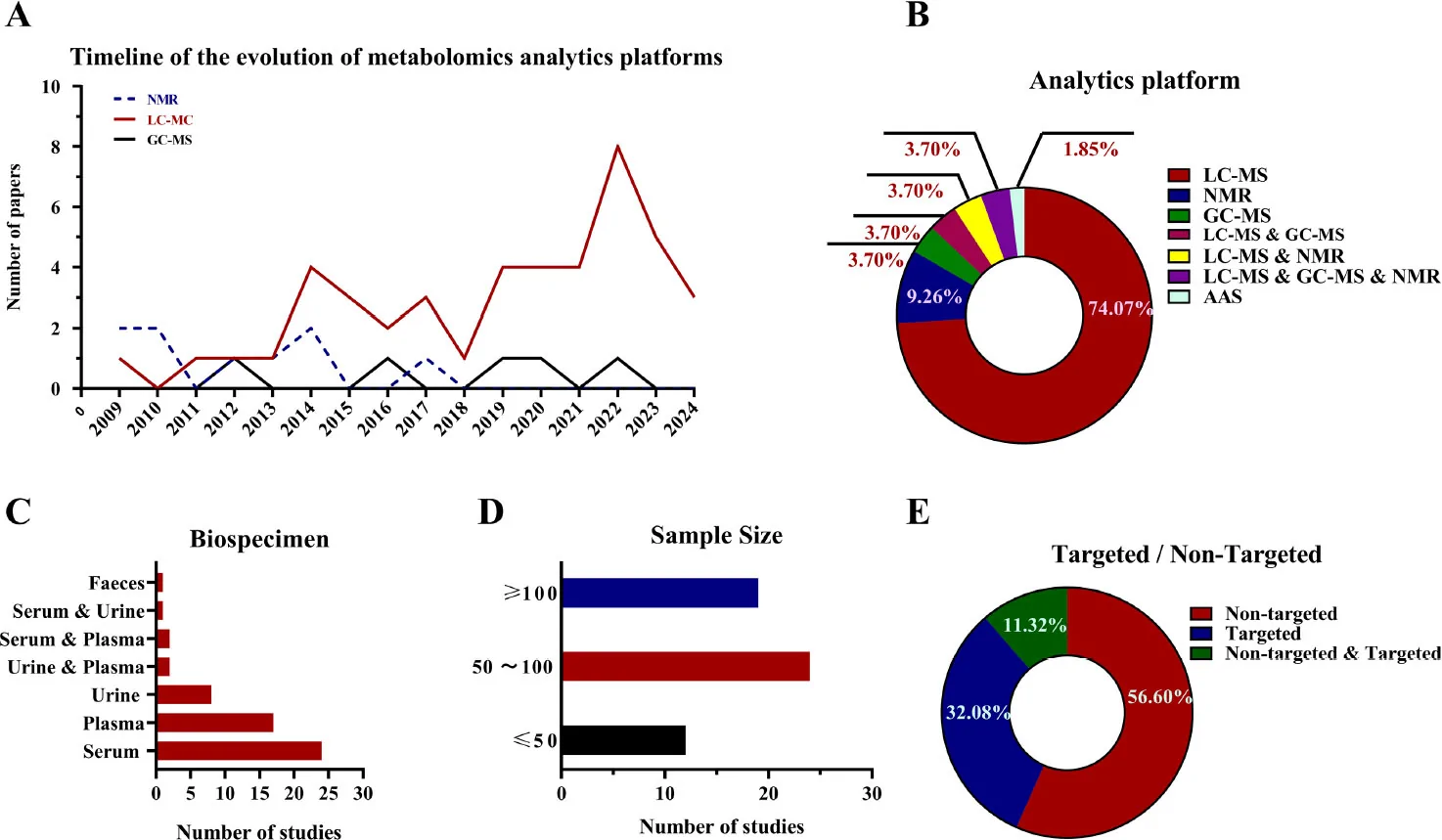
Characteristics of the included studies. (A-B) Number of included studies according to analytics platform. (C) Number of included studies according to biosample type. (D) Number of included studies according to sample size. (E) Number of included studies according to targeted/non-targeted.

Based on the characteristics of the 55 studies, including analysis platform, sample type, sample size, and the distinction between targeted and untargeted detection methods, we conducted a comprehensive statistical analysis (Figure 2). As depicted in Figure 2A and Figure 2B, the evolution of metabolomics detection technology can be traced over time, transitioning from NMR to GC-MS, and subsequently to LC-MS. Since 2014, LC-MS has emerged as the predominant detection platform in metabolomics research. Among the 55 studies reviewed, 41 employed LC-MS technology. Figure 2C illustrates that the primary biological samples utilized in metabolomics studies were blood samples, encompassing both serum and plasma, as well as urine. Specifically, 46 studies employed blood samples, 8 utilized urine samples, and 1 employed fecal samples. Within the blood samples, 27 studies analyzed serum, while 22 focused on plasma. Regarding sample size, 43 studies included more than 50 individuals, with a significant concentration on sample sizes ranging from 50 to 100 individuals, as exemplified in Figure 2D. Furthermore, of the metabolomics studies included, 30 were designated as targeted assays, 17 as non-targeted assays, and 4 employed a hybrid approach, integrating both non-targeted and targeted assays, as illustrated in Figure 2E.

### Geographic distribution of studies and DILI drugs in focus

**Figure 3:**
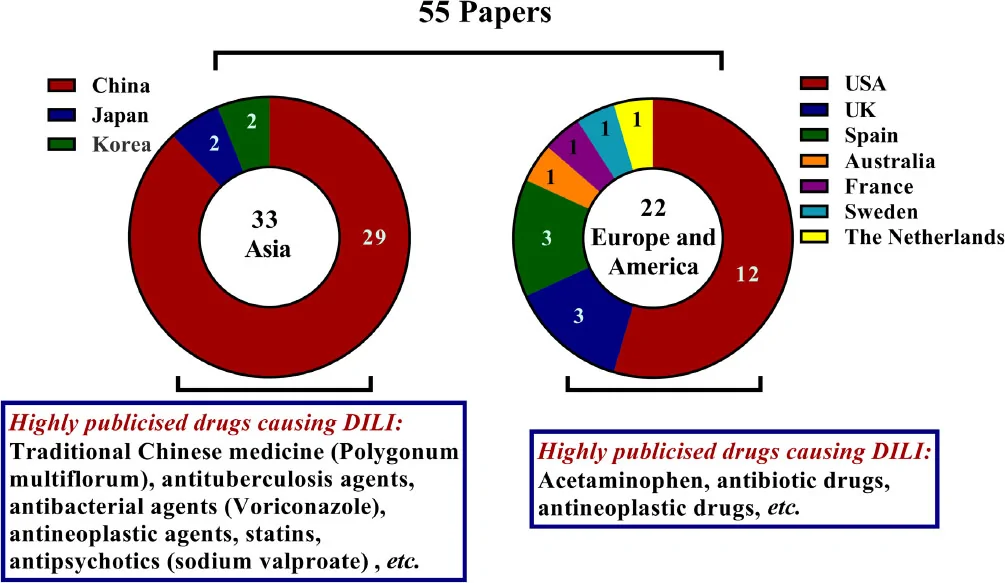
Distribution of countries and highly publicised drugs in included studies.

This systematic analysis synthesized the current research landscape regarding DILI, with specific emphasis on both the implicated drugs and their geographical distribution (encompassing Asia, Europe, and the America), as investigated through metabolomics techniques (Figure 3). It delineates the regional distribution, with 33 studies emanating from Asian countries, notably with 29 studies originating from China, and 22 studies from Europe and the United States, including 12 from the United States. The etiology of DILI in the Asian demographic was attributed to a spectrum of medications, including traditional Chinese medicine such as PM, herbal remedies containing pyrrolizidine alkaloids, anti-tuberculosis agents, antimicrobials like voriconazole, antineoplastic compounds, statins, and antiepileptic medications such as sodium valproate. Conversely, in the European and American populations, DILI was predominantly associated with acetaminophen, a range of antimicrobials, and antineoplastic drugs.

### Predictive and diagnostic potential of DILI metabolic markers

In the 55 studies, a comprehensive screening was conducted to identify metabolic markers exhibiting significant alterations. Among these, 32 studies specifically evaluated the predictive and diagnostic utility of individual metabolic markers or their combinations in the context of DILI, as detailed in Table 1. Each study calculated the area under the receiver operating characteristic curve (AUC) for these markers or their combinations. All reported metabolomic biomarkers exhibited AUC values > 0.6, supporting their diagnostic potential through preliminary validation. These investigations leverage advanced analytical platforms (serum, plasma, urine metabolomics; lipidomics; microbiome analysis) combined with machine learning (ML) to elucidate mechanisms and enhance clinical risk assessment. Soga^[68]^ identified *γ*-glutamyl dipeptides as potential serum biomarkers for liver disease differentiation. Utilizing multivariable logistic regression, the area under the curve values in training and independent validation data were 0.952 and 0.967 in healthy controls, 0.817 and 0.849 in DILI. Wang^[41]^ investigated ATB-DILI mechanisms through integrated urine metabolomics and microbiome profiling. Four distinct ML prediction models demonstrated robust performance, with training and validation AUCs consistently exceeding 0.85. Zhao^[53]^ systematically analyzed serum metabolites and gut microbiota in DILI patients. An RF model incorporating arachidonic acid (AdA) and aspartate (Asp) effectively predicted DILI chronicity risk, achieving high training (AUC = 0.889) and validation (AUC = 0.888) accuracy. Collectively, integrated pharmacometabonomics, through multi-omics data convergence, demonstrates robust capability in delineating DILI-specific metabolic perturbations and identifying sensitive biomarkers.

**Table 1:** Potential of metabolic markers for the prediction or diagnosis of DILI

Number	Reference	Medicine	Outcome	Sample type	Potential Biomarker(s) or Biomarker Panel	AUC	Sensitivity	Specificity	Clinical significance
1	Angela L. Chiew^[16]^	APAP	ALT > 1000 U/L	Serum	Combination of 3 markers (APAP ratio, APAP- Cys and ALT)	0.950	–	–	Cytochrome P450 and sulfate metabolites may enable early identification of high-risk liver injury patients on APAP treatment.
2	Brandon J. Sohn^[17]^	APAP	ALT > 60 IU/L	Serum	Combination of 3 metabolites (ALT, ALKP, and ornithine)	0.799	–	–	Pre-treatment metabolites can predict patient risk for transient increases.
3	James W Dear^[18]^	NAC protection to APAP overdose patients	APAP-ALT	Plasma	Purine metabolite: Xanthine Xanthine/total Sum of APAP metabolites panel and xanthine/total	0.91 0.90 0.98	–	–	Plasma purine metabolites may be useful biomarkers for early prediction of APAP induced ALI.
4	Sudeepa Bhattacharyya^[21]^	APAP	Hospitalized for APAP overdose	Serum	APAP overdose vs. control lysoPC-C26:1 (cut-point of 0.9 uM)	1.000	1.000	1.000	LysoPC-C26:1 was identified as a strong candidate biomarker for APAP toxicity.
5	Laura James^[22]^	APAP	APAP-DILI	Serum	Taurodeoxycholic acid Glycodeoxycholic acid	0.92 0.80	0.89 0.67	0.84 0.87	In children with acetaminophen overdose, conjugated bile acids elevation correlates with acetaminophen metabolism and liver injury indicators.
6	Benjamin L. Woolbright^[25]^	APAP	APAP-ALF	Serum	Predicting death on day of study admission: Glycodeoxycholic acid	– 0.7	–	–	Glycodeoxycholic acid has potential value as a prognostic indicator in the APAP-ALF.
7	JH Winnike^[28]^	APAP	APAP Induced Hepatotoxicity	Urine	The endogenous components for days 5 and 6 The endogenous components for days 9 and 10	0.714 0.771	–	–	Metabolome perturbations primarily differentiate APAP-induced hepatotoxicity responders from nonresponders.
8	Aizhen Xiong^[31]^	Pyrolizidine alkaloids from herbal product	PAS-HSOS	Serum	PA-HSOS *vs*. healthy: CA species Glycocholic acid Taurocholic acid Cholic acid	0.968 0.966 0.964 0.833	0.839 – – –	0.966 – – –	Increased levels of cholic acid species can serve as diagnostic biomarkers in PA-HSOS.
9	Yinghuan Hu^[32]^	Polygonum multiflorum	PM-DILI	Plasma	Hypoxanthine LysoPC(P-16:0/0:0) Taurochenodeoxycholic acid	0.974 1.000 0.974	1.000 1.000 1.000	0.846 1.000 0.846	Selected Polygonum multiflorum liver injury biomarkers facilitate early clinical diagnosis.
10	Ying Huang^[33]^	PM	PM-DILI	Plasma	Combination of the ratio of P-cresol sulfate/ phenylalanine and the ratio of inosine/bilirubin	0.931	0.923	0.889	Metabolomic markers may distinguish PM-DILI from ALI and HBV, and have potential clinical diagnostic value.
11	Le Zhang^[34]^	PM	PM-DILI	Serum	PE 22:6 indole-5,6-quinone 2E-tetradecenoyl-CoA crotonoyl-CoA phenyllactic acid phosphoribosyl-ATP	0.939 0.917 0.911 0.933 0.906 0.900	–	–	Metabolomics approach may predict risk of PM induced liver injury.
12	Meng Tian^[36]^	Cadmium	Cd-induced liver injury	Serum	Glycoursodeoxycholic acid Glycolithocholic acid Taurodeoxycholic acid Tauroolithocholic acid	0.868 0.776 0.826 0.731	0.875 0.788 0.733 0.945	0.761 0.663 0.750 0.489	Serum Bile acids were shown to be valuable in assessing cadmium-induced liver injury and cadmium exposure in humans.
13	Yi-Hsuan Chiang^[39]^	Voriconazole	DILI	Plasma	Combining glycocholate with voriconazole trough concentrations glycocholate *α*-ketoglutarate	0.827 0.795 0.696	0.833	0.673	The panel integrating glycocholate with voriconazole trough concentrations has great potential for identifying voriconazole-associated hepatotoxicity.
14	Ming-Gui Wang^[40]^	Anti-tuberculosis drug	ATB-DILI	Plasma	30 important variables for the training set / the validation set:	0.79 / 0.79	—	—	This study demonstrated the potential markers for the early prediction of ATB-DILI
15	Ming-Gui Wang^[41]^	Anti-tuberculosis drug	ATB-DILI	Urine	Machine learning models for the training set / the validation set :	0.98 / 1.00	—	—	Metabolomic and microbiome changes from the host can be used to identify and predict an individual's susceptibility to ATB-DILI.
16	Shouquan Wu^[42]^	Anti-tuberculosis drug	ATDH	Urine	Differential metabolites	0.962	—	—	Urinary metabolomic markers for ATDH diagnosis and prognosis are identified.
17	Shin-Lun Wu^[45]^	Voriconazole	Voriconazole-induced hepatotoxicity	Plasma	*α*-Ketoglutarate voriconazole trough concentrations (*α*-ketoglutarate + glycocholate + *β*-N-acetylglucosamine)	0.750 0.868 0.749 0.932	—	—	These potential biomarkers may predict voriconazole-induced hepatotoxicity.
18	Kaini He^[51]^	Not mentioned	DILI	Faeces	Cortodoxone Prostaglandin 11 Bioyolo PGE2 Anacardic acid	0.867 0.912 0.913 0.856	—	—	Differential metabolites in DILI may help in the diagnosis or prediction of DILI.
19	Francisco Andujar-Vera^[52]^	Horsetail (Equisetum), Green anise, Amoxicillin-clavulanic acid, Methotrexate, Ibuprofen, Isotriazole, Acyclovir, Cotrimoxazole, Cloxacillin, Amoxicillin, Voriconazole, Zonisamide, Meropenem, Propranolol, Amiodarone, Rifampicin	DILI	Plasma	Combination of 5 metabolites (lysophosphatidylcholine (16:1/0:0), glycocholic acid, taurocholic acid, hydroxydecanoylcarnitine and octanoylcarnitine)	0.817	—	—	This study sought pediatric plasma biomarkers for hepatotoxicity characterization.
20	Shuang Zhao^[53]^	Conventional drugs and herbs	DILI	Serum	Discriminate chronic (DILI.chr) from recovered (DILI.rec): The training set The validation set The panel of AdA and Asp	0.889 0.888 0.850	0.737 0.714	0.927 0.913	It identifies DILI severity and chronicity-related metabolites, offering potential early biomarkers for chronic DILI intervention.
21	Xian He^[54]^	Chronic DILI patients	Chronic DILI to progress into liver fibrosis and even cirrhosis	Serum	Fibrosis eigenmetabolite Advanced-fibrosis eigenmetabolite	0.753 0.944	—	—	The unique metabolomic fingerprints associated with chronic DILI fibrosis have potential clinical diagnostic and prognostic significances.
22	Yan-zhong Han^[55]^	Not mentioned	AIH, DILI^Ab+^ and DILI^Ab-^	Serum	74 fingerprint metabolites	0.891	—	—	Offering a clinical screening reference for autoimmune-featured DILI, addressing the current empirical screening limitation.
23	Xinbei Tian^[56]^	Biliary atresia patients	Biliary atresia	Plasma	D-2-hydroxyglutarate	0.890	—	—	D-2-HG may represent a novel noninvasive diagnostic biomarker and a potential therapeutic target for infants with BA.
24	Zhongyang Xie^[57]^	Polygonum Multiflorum, Fructus Psoraleae, Anti-Tuberculosis, cephalosporin, Azithromycin, Amoxicillin	DILI (mild DILI, moderate DILI and severe DILI)	Serum	Non-severe DILI vs. severe DILI: Glycoch enodeoxycholic acid Taurochenodeoxycholic acid Nor-Cholic acid Combination of the above 3 metabolites	0.856 0.792 0.753 0.895	—	—	Three metabolites, as independent risk factors, differentiate severe DILI patients and may predict severity.
25	Shuai-shuai Chen^[58]^	Chronic DILI patients	Chronic DILI with or without cirrhosis	Serum	APRI + metabolic fingerprint APRI	0.914 0.573	—	—	Metabolomic fingerprints characterize chronic DILI stages, providing clinical diagnosis and monitoring references for associated cirrhosis development.
26	Kosuke Saito^[60]^	Loxoprofen, antibacterial agents, antineoplastic agents, anti-inflammatory and antirheumatic products, psychotropics	DILI	Plasma	The mixed-type DILI vs. healthy subjects: (Cer(d34:1; d18:1/16:0) Cer(d36:1; d18:1/18:0) GM3(d34:1) + O) The cholestatic-type DILI vs. healthy subjects: PC(30:0; 14:0/16:0) PC(31:0; 15:0/16:0) PC(32:1; 16:0/16:1) PC (33:1; 15:0/18:1, 16:0/17:1) PI(38:3) PI(38:4; 18:0/20:4) SM(d40:1; d18:1/22:0)	> 0.8 > 0.8 > 0.8 > 0.8 > 0.8 > 0.8 > 0.8	—	—	Identify DILI lipid biomarkers for understanding diverse DILI pathophysiology.
27	Zhenhua Ma^[61]^	Traditional Chinese medicine, Analgesic-antipyretic drugs, Antituberculosis drugs, Antibiotic drug, Weight-reducing medicine, Antihypertensive drugs, Lipid-lowering drugs, Tibetan medicine, Healthcare products.	Mild DILI and Severe DILI	Serum	Glycocholic acid Taurocholic acid Tauroursodeoxycholic acid Glycoch enodeoxycholic acid Glycochenodeoxycholic sulfate Taurodeoxycholic acid Deoxycholic acid Lithocholic acid Chenodeoxycholic acid	0.978 0.985 0.909 0.954 0.946 0.976 0.77 0.66 0.67	—	—	Bile acids may be potential biomarkers for early diagnosis of DILI and DILI severity
28	Zhongyang Xie^[62]^	Clarithromycin, Cephalosporin, Amoxicillin, Piperacillin-tazobactam, Capecitabine, Sodium valproate, Ibuprofen, TNF-α antagonist, Isoniazid/Rifampicin/Pyrazinamide, Acyclovir, Rabeprazole, Rabies vaccines, Phenobarbital.	Severe DILI and Non-severe DILI	Serum	5 cytokines and 31 metabolites	0.983	-	-	These biomarkers have potential as indicators of DILI severity.
29	Xiaohui Wang^[63]^	Not mentioned	ALD, DILI and ALD-DILI	Serum	DILI vs. ALD-DILI: phosphatidate Phosphatidylethanolamine Tauro-lithocholate 3-Ketolactose Phosphatide/3-Ketolactose Phosphatide/taurolithocholate	>0.8 >0.8 >0.8 >0.8 0.886 0.918	-	-	Candidate biomarkers have better diagnostic efficacy in differentiating DILI from ALD-DILI.
30	Luge Wei^[64]^	Not mentioned	Liver cirrhosis-DILI and chronic-DILI	Serum	Liver cirrhosis-DILI vs. Chronic-DILI: phosphatidylcholine lysoPC (18:1 (9Z)) creatine taurochenodeoxycholic acid taurocholic acid phosphatidylcholine/ lysoPC (18:1 (9Z))	>0.6 >0.6 >0.6 >0.6 0.867	-	-	Screened biomarkers may allow early detection of cirrhotic patients with chronic DILI.
31	Jiabo Wang^[66]^	Not mentioned	AIH and DILI	Plasma	AIH patients *vs*. DILI patients: nine screened metabolic markers	0.954	1.000	0.933	Plasma metabolites can be used to differentiate AIH patients from PBC, DILI, and PBC/AIH overlap patients.
32	Tomoyoshi Soga^[68]^	Not mentioned	DILI	Serum	The training / the validation: the model for controls the model for DILI	0.952 / 0.967 0.817/0.849	-	-	A specific set of γ-glutamyl dipeptides, transaminases, and methionine sulfoxide serves as multiple biomarkers for rapid liver disease screening across types and stages.

AA/PAP: Acetaminophen; ALT: Alanine Aminotransferase; ALP: Alkaline Phosphatase; PAS-HSOS: pyrrolizidine alkaloids induced-Hepatic sinusoidal obstruction syndrome; PM-DILI: polygonum multiflorum-induced liver injury; PE: phosphatidylethanolamine; ATB: anti-tuberculosis drug; ATDH: anti-tuberculosis drug-induced hepatotoxicity; DILI: Drug-Induced Liver Injury; DILIAb+: DILI with autoantibodies; DILIAb-: DILI without autoantibodies; APRI: aspartate aminotransferase-to-platelet ratio index; Cer: ceramide; PC: phosphatidylcholine; PI: phosphatidylinositol; SM: sphingomyelin; ALD: alcohol-associated liver diseases; AIH: autoimmune hepatitis.

Subsequently, we compiled a summary of the metabolic markers that were frequently assessed, as delineated in Table 2. These markers are ranked according to their frequency of occurrence, with glycocholic acid (GCA), taurochenodeoxycholic acid (TCDCA), taurocholic acid (TCA), creatine, glycochenodeoxycholic acid (GCDCA), taurodeoxycholic acid (TDCA), glycocodeoxycholic acid (GDCA), and alpha-ketoglutaric acid being the most frequently observed. With the exception of creatine and alpha-ketoglutaric acid, the remaining six high-frequency markers are categorized as bile acids. Notably, GCA was the most frequently reported, appearing seven times across the studies. It was evaluated and validated across serum, plasma, and urine samples, consistently demonstrating an increasing trend.

**Table 2:** High-frequency metabolic biomarkers of DILI

Metabolic biomarkers	Frequency	Sample	References
Glycocholic acid	7	Serum Plasma Urine	[31],[58],[61] [39],[45],[52] [41]
Taurochenodeoxycholic acid	6	Plasma Serum	[32] [57],[58],[61],[62],[64]
Taurocholic acid	4	Serum Plasma	[31],[64] [52],[61]
Creatine	4	Urine Serum Plasma	[41],[42] [64] [66]
Glycochenodeoxycholic acid	3	Serum	[54],[57],[62]
Taurodeoxycholic acid	3	Plasma Serum	[56] [22],[61]
Glycodeoxycholic acid	2	Serum Serum and Plasma	[22] [25]
Alpha-Ketoglutaric acid	2	Plasma	[39],[45]

### Analysis of significantly altered metabolic pathways in DILI

**Figure 4:**
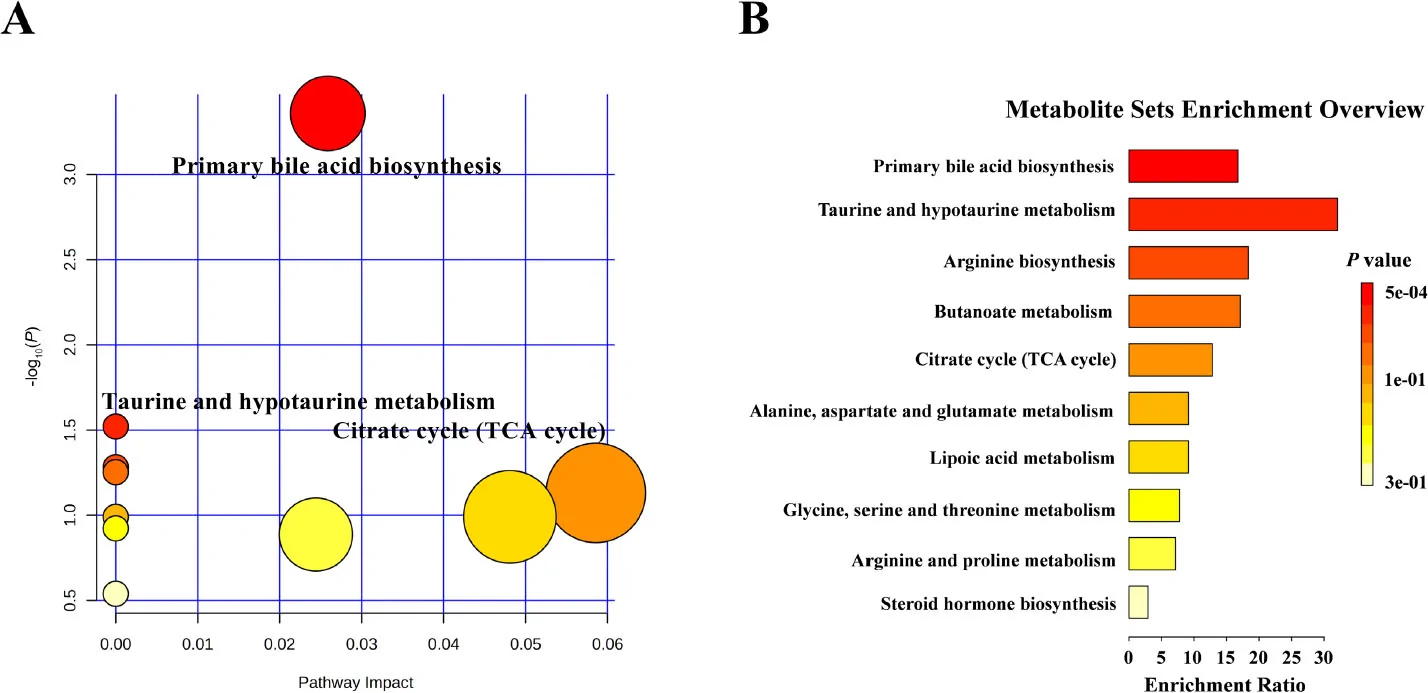
Metabolic pathway analysis in DILI. (A) Overview of pathway Analysis. (B) Overview of enrichment analysis. DILI: drug-induced liver injury.

The 55 studies reviewed identified a spectrum of metabolic markers that exhibited significant alterations. Utilizing these markers, we have delineated the metabolic pathways that were consistently and significantly perturbed across the studies. A summary of the high-frequency metabolic pathways that were significantly altered is presented in Table 3. The pathways that were most frequently and significantly altered, ranked by their significance, include primary bile acid biosynthesis, fatty acid biosynthesis, glycerophospholipid metabolism, and the Tricarboxylic Acid Cycle (TCA), each noted in a minimum of eight studies. Additionally, pathway enrichment analysis of the top eight DILI markers, as shown in Figure 4 and Table 4, confirms the significant association of the primary bile acid metabolic pathway with DILI.

**Table 3:** Altered metabolic pathways of DILI

Metabolic pathways	Frequency	References
Bile acid metabolism	19	[22], [25], [27], [31], [33], [45], [50], [52], [53], [54], [55], [57], [58], [59], [61], [62], [63], [64], [67]
Fatty acid biosynthesis	16	[23], [24], [27], [31], [33], [34], [35], [40], [48], [53], [54], [55], [60], [61], [62], [67]
Glycerophospholipid metabolism	11	[21], [32], [33], [34], [35], [50], [55], [59], [62], [63], [67]
Tricarboxylic acid cycle	8	[14], [19], [26], [44], [46], [55], [56], [58]
Glutathione metabolism	6	[14], [17], [19], [28], [42], [68]
Arginine and proline metabolism	6	[19], [41], [42], [46], [53], [58]
Sphingolipid metabolism	5	[31], [32], [33], [34], [54]
Purine metabolism	5	[18], [37], [45], [46], [65]
Oxidative stress responses	4	[27], [45], [47], [68]
Glycine, serine and threonine metabolism	3	[19], [42], [63]
Pentose phosphate pathway	3	[44], [46], [54]
Urea cycle pathways	3	[14], [17], [37]

DILI: drug-induced liver injury.

**Table 4:** Results of the pathway analysis

Pathway name	Raw *P* value	Impact
Primary bile acid biosynthesis	0.00043899	0.02587
Taurine and hypotaurine metabolism	0.030139	0.00000
Arginine biosynthesis	0.052243	0.00000
Butanoate metabolism	0.055886	0.00000
Citrate cycle (TCA cycle)	0.073926	0.05856
Lipoic acid metabolism	0.10219	0.00000
Alanine, aspartate and glutamate metabolism	0.10219	0.04808
Glycine, serine and threonine metabolism	0.11949	0.00000
Arginine and proline metabolism	0.12974	0.02442
Steroid hormone biosynthesis	0.28929	0.00000

TCA: tricarboxylic acid.

**Figure 5:**
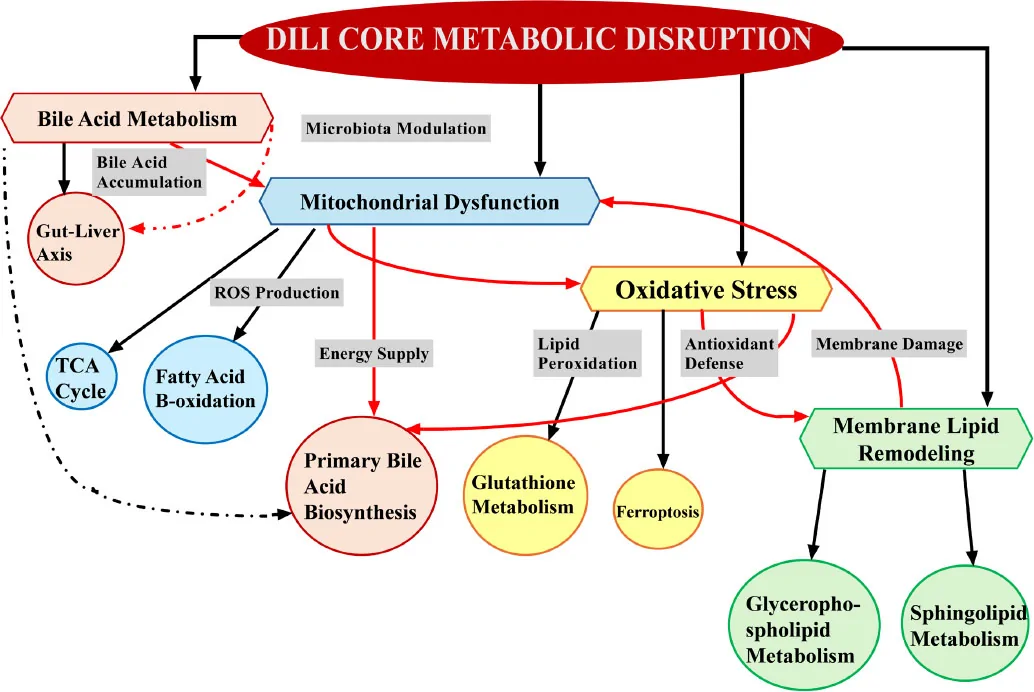
Diagram of core metabolic disturbances in DILI. The size of each circle represents the extent of alteration for a specific pathway in DILI, with larger circles indicating more significant changes. Pathways are color-coded: red for bile acid-associated pathways, green for membrane lipid-related pathways, blue for mitochondrial function pathways, and yellow for oxidative stress pathways. Solid thick arrows denote primary pathological mechanisms, while thin dashed arrows represent secondary regulatory relationships. DILI: drug-induced liver injury.

Building upon these findings, integrating all significantly altered metabolic pathways associated with DILI identified in the included literature, we visualized the metabolic disturbances in DILI as a metabolic pathway network diagram (Figure 5). Consequently, the core metabolic disturbances in DILI arise from a combination of disrupted bile acid homeostasis, mitochondrial energy crisis, redox imbalance, and membrane lipid remodeling.

## Discussion

In this systematic review, we conducted an exhaustive analysis of metabolomics studies pertaining to DILI. Our objective was to identify metabolic pathways and markers that exhibit significant alterations. We compiled a list of frequently altered metabolic pathways and evaluated eight prominent metabolic markers, subsequently conducting a comprehensive pathway analysis. The findings indicated that bile acid metabolism, lipid metabolism, and TCA cycle were notably disrupted in DILI, implicating a complex metabolic dysregulation in affected patients. Bile acids, particularly GCA, emerged as the most promising metabolic markers for DILI.

### Bile acid metabolism and DILI

Bile acids are pivotal metabolites within the body, significantly influencing lipid digestion and absorption as well as regulating energy metabolism. The presence of drugs, their metabolites, or exogenous chemical toxins can disrupt the synthesis, secretion, or elimination of bile acids, potentially leading to their accumulation in the liver and the onset of cholestasis. For instance, specific pharmaceuticals may impede the function of hepatic transporter proteins that are crucial for bile acid excretion, such as multidrug resistance-associated protein 2 (MRP2) and the bile salt efflux pump (BSEP), thereby obstructing bile acid secretion. This disruption can precipitate hepatic dysfunction, including hepatocellular injury, cholestasis, and potentially advancing to hepatic fibrosis and cirrhosis.^[10, 69, 70]^ DILI induces alterations in the metabolic profile of bile acids. Typically, bile acids are predominantly confined to the hepatic-intestinal circulation, with minimal concentrations detectable in systemic circulation and urine. However, in the event of impaired bile acid excretion due to DILI, there is a marked increase in bile acid levels within the liver, blood, and urine.^[71, 72, 73]^ Consequently, the quantification of aberrant bile acid concentrations (both conjugated and unconjugated) and other biliary components, such as phospholipids, may serve as promising biomarkers for the identification of cholestatic DILI through metabolomic profiling.^[74, 75]^

In the literature reviewed for this study, 19 studies demonstrated a significant association between bile acid metabolism and DILI. A subset of these studies indicated that bile acid levels possess predictive power for the early identification and diagnosis of DILI,^[22,31,32,36,52,64]^ while another subset corroborated a close relationship between bile acid levels and the severity, progression, and prognosis of DILI.^[25, 53, 57]^ Andújar-Vera *et al*.^[52]^ confirmed an increase in bile acids in pediatric patients with drug-induced hepatotoxicity, identifying GCA and TCA as diagnostic markers for the development of DILI. Shuang Zhao *et al*.,^[53]^ through metabolomic analysis, revealed a strong correlation between bile acid levels and the severity of DILI. Notably, patients with DILI exhibited an elevated ratio of primary to secondary bile acids in their serum. Tian *et al*.^[36]^ investigated the role of bile acids in cadmium-induced liver injury, also demonstrated that GUDCA, GLCA, TDCA, and TLCA are clinically valuable for assessing both cadmium-induced liver injury and exposure to cadmium.

Although the diagnosis and classification of DILI currently rely on abnormal liver biochemical test results (serum levels of alkaline phosphatase [ALP] and aspartate aminotransferase [AST]), metabolomics may offer more specific information. Individual or combined bile acids, or in conjunction with other biomarkers, hold the potential to effectively predict and diagnose DILI. However, the validation of biomarkers is limited due to the small sample sizes, heterogeneity in target populations, and variability in detection platforms and methods used in related studies. Therefore, further research, such as studies involving independent external cohorts with larger sample sizes, is necessary to obtain reliable bile acid biomarkers.

### Lipid metabolism and DILI

Alterations in bile acid metabolites are notably significant; however, lipid changes also enhance our understanding of the pathogenesis of DILI. In the literature reviewed for this study, fatty acid biosynthesis (*n* = 16), glycerophospholipid metabolism (*n* = 11), and sphingolipid metabolism (*n* = 5) have consistently been reported to be closely associated with DILI. Lipids, as integral constituents of the cell membrane, are essential for preserving cellular architecture, functionality, signal transduction, and energy storage.

Phosphatidylcholine (PC) and sphingomyelin (SM) are primarily situated in the exoplasmic leaflet of the cell membrane, while ceramide (Cer) is typically found in the cytoplasmic leaflet or functions as a signaling molecule within cellular signal transduction pathways. Disruptions in lipid metabolism could be fundamental to the pathogenesis of intrahepatic cholestasis. PC, a structural lipid, has been observed to decrease in DILI patients. Dysfunctional PC secretion into bile, coupled with a marked reduction in biliary phospholipid concentrations, can result in damage to the canalicular membrane of cholangiocytes, leading to biliary pathologies and consequent cholestasis. Sphingolipids, ubiquitous structural components of eukaryotic cell membranes, serve as regulators of hepatic homeostasis, modulators of liver regeneration, and biomarkers of hepatocyte injury, thereby governing a multitude of biological processes. A robust association exists between the pathogenesis of DILI and perturbations in sphingolipid homeostasis, with a particular emphasis on the roles of Cer and SM.^[76]^

Wang *et al*.^[40, 41]^ conducted a metabolomic study on patients with liver injury induced by anti-tuberculosis drugs, revealing alterations in lipid metabolic components in both plasma and urine samples, with significant changes observed in the pathways of nicotinic acid and nicotinamide metabolism. Quintás *et al*.^[59]^ identified free and conjugated bile acids, along with glycerophospholipids, as one of the most critical metabolic categories characterizing DILI phenotypes in a cohort of 79 patients. Integrating metabolomic data into predictive models can elucidate DILI phenotypes, the severity of liver injury, and its progression, thereby complementing the information on disease progression that is not provided by ALT and ALP levels. Zhang *et al*.^[34]^ focused on a population with idiosyncratic DILI caused by PM. They discovered significant serum metabolomic differences between susceptible and tolerant groups, particularly in glycerophospholipid metabolism, sphingolipid metabolism, and fatty acid metabolism. The areas under the ROC curves for lipid metabolites PE 22:6, crotonoyl-CoA, and 2E-tetradecenoyl-CoA were all greater than 0.9. Consequently, lipid metabolites can also be considered as potential diagnostic targets for DILI.

### TCA cycle and DILI

TCA cycle, also known as the citric acid cycle or Krebs cycle, is a central metabolic pathway that occurs within the mitochondria, converting organic substrates into carbon dioxide and water through a series of enzymatic reactions. This process is instrumental in generating adenosine triphosphate (ATP), the primary energy currency of the cell. During DILI, drugs or their metabolites can directly impair hepatocytes and disrupt mitochondrial function, consequently hindering the normal operation of the TCA cycle. Furthermore, DILI can induce oxidative stress, leading to an overproduction of reactive oxygen species (ROS). These ROS can inflict damage on mitochondrial DNA, proteins, and lipids, which in turn consequently impair the enzymatic activities within the TCA cycle and the efficacy of the electron transport chain.^[77]^ Additionally, DILI may disrupt the supply of substrates necessary for the TCA cycle by influencing specific metabolic pathways. For instance, the *β*-oxidation of fatty acids could be compromised, thereby affecting the availability of substrates for the TCA cycle. An impairment in fatty acid metabolism may result in a scarcity of TCA cycle substrates, adversely impacting energy production. In the studies referenced,^[14, 19]^ it was noted that energy metabolic pathways, particularly the TCA cycle, are significantly disrupted in patients exhibiting hepatotoxicity due to acetaminophen overdose.

### APAP-induced DILI

The hepatotoxic progression of acetaminophen (APAP) is characterized by a well-defined trajectory of exogenous metabolite dysregulation. Initially, detoxification failure occurs as therapeutic doses of APAP are predominantly metabolized *via* glucuronidation (APAP-Glu, 55%) and sulfation (APAP-Sul, 30%), while a minor fraction undergoes CYP2E1/3A4-mediated oxidation to form the toxic metabolite NAPQI (5%–10% of the dose). Upon overdose, a toxic shift ensues, with NAPQI overload depleting hepatic glutathione, thereby suppressing the formation of APAP-Cys and APAP-Mer (< 4% of metabolites) and promoting covalent binding to cellular proteins (elevated APAP-adducts). This sets the stage for toxicity amplification, as mitochondrial dysfunction induces a burst of ROS, further exacerbating liver injury. As the condition progresses, dysregulated one-carbon metabolism leads to glycine depletion, impairing glutathione (GSH) regeneration and triggering epigenetic alterations. Importantly, total Cytochrome P450 (CYP) metabolite levels (APAP-Cys + APAP-Mer) exceeding 36.8*μ*mol/L predict hepatotoxicity with over 90% sensitivity, while suppressed APAP-Sul levels signify impaired detoxification pathways. This sequential trajectory, encompassing detoxification failure, oxidative burst, inflammatory cascade, and repair impairment, offers a robust framework for diagnostic stratification and targeted therapeutic interventions in APAP-induced hepatotoxicity.

Meanwhile, the endogenous metabolic network experiences significant disruption, with several key metabolic pathways being affected in APAP-DILI. The glutathione-urea cycle axis is perturbed, characterized by the collapse of glutathione (GSH) synthesis (evidenced by decreased glycine and 5-oxoproline levels) and disruption of the urea cycle (marked by increased ornithine and decreased citrulline levels), which collectively exacerbate oxidative stress. Additionally, mitochondrial energy failure is observed, with inhibition of the tricarboxylic acid (TCA) cycle leading to reduced citrate levels and accelerated glycolysis, correlating with ATP depletion. Bile acid homeostasis is also disrupted, with accumulation of hydroxylated bile acids (*e.g*., GDCA, TDCA) and a decrease in protective bile acids (*e.g*., CA/TCA). These findings highlight that glutathione metabolism, bile acid homeostasis, and the TCA cycle are the core disrupted networks in APAP-DILI. Furthermore, metabolites such as glycine, acylcarnitines, and lysophosphatidylcholines (LysoPCs) have emerged as potential prognostic indicators, underscoring the importance of metabolomics in elucidating the pathophysiology of APAP-DILI.

Overall, while variations in individual metabolite concentrations may not be uniformly reproducible across different studies, the disruptions in metabolic pathways, as delineated by functional and pathway analyses in DILI, exhibit a consistent and reproducible pattern across investigations. These findings align with the established pathophysiological progression of DILI. In addition, we systematically classified and summarised 55 studies according to specific drugs and co-medications associated with DILI. Our attempts to identify specific biomarkers of DILI in cohorts exhibiting similar metabolic profiles due to the same drug or concomitant administration of the same drugs were less than expected. Consistent endogenous metabolic markers have not been identified, except in single-drug studies such as the APAP-induced DILI study,^[15,16,23,27,28]^ where drug metabolite products were found to be strongly associated with DILI severity. This lack of consistency may be intrinsically linked to the considerable heterogeneity observed in DILI metabolomics studies.

### Limitations of DILI metabolomics studies

DILI metabolomics studies face certain limitations. Firstly, the majority of subjects assessed for hepatic function (HF) metabolic markers were recruited in China, which may introduce constraints regarding the generalizability of the study population. Bile acids, as the most promising DILI biomarkers, necessitate further validation in multicenter, large-sample cohorts across diverse global populations. Concurrently, bile acid production and metabolism are influenced by various factors, including diet, immune status, and lifestyle. The types of biological samples appropriate for metabolomics assays and the optimization of sampling conditions require further validation. Secondly, metabolomic marker research for DILI remains in its nascent phase. Additional cohort studies and methodological validations are imperative before the translation of these findings into clinical practice can be realized. Robust external cohorts or complementary animal and cellular experiments are essential to validate, expand, and enrich the current understanding of DILI. Lastly, the integration of metabolomics with other ‘omics’ disciplines, such as genomics, transcriptomics, and proteomics, may facilitate a more comprehensive understanding of the intricate mechanisms underlying DILI.

## Conclusion

In conclusion, this systematic review of metabolomic studies investigating DILI consistently identifies perturbations in bile acid metabolism as exhibiting the most pronounced and robust association with DILI pathogenesis, highlighting its potential as a mechanistic biomarker. Furthermore, it has been established that lipid metabolism and the TCA cycle are also markedly disrupted in patients afflicted with DILI. The metabolic biomarkers identified in these studies indicate the promising clinical utility of metabolomics in the prognostic and diagnostic realms of DILI. Consequently, there is a clear imperative to promote more comprehensive and sophisticated metabolomic research endeavors. Such studies are essential to reveal more about the pathogenesis of DILI and to enable accurate diagnosis and prediction of DILI.

## Supplementary Material

Supplementary Material Details
