# Supplementary material for: Application of metabolomics in drug-induced liver injury: A systematic review
Source: J Transl Int Med. 2026 Aug 12;14(4):547–62. doi: 10.1515/jtim-2026-0056 (PMC13508834; doi:10.1515/jtim-2026-0056)
Supplement: Supplementary file 1 [file jtim-2026-0056_sm.pdf]

Supplementary materials

Supplementary Table S1: Characteristics of Acetaminophen-related liver injury studies (*n* = 17)

First Author (Year) (Refs.)	Country	Medicine	Outcome	Biochemical test indicators	Case / Control	Analytic Platform	Targeted/ Non-targeted	Sample type	Up-regulated biomarkers	Down-regulated biomarkers
Andrew A. Monte (2023) [14]	USA	APAP	ALT elevation	ALT	192/n*	UPLC–MS/MS	Non-targeted	Plasma	Significant metabolite-ALT elevation associations: 3-Oxalomalate, Allantoate, Dimethylglycine, Diphosphate, L-Carnitine, L-Proline, Maltose and Ornithine.	None[#]
Eva Gorrochategui (2023) [15]	France	APAP	Acute APAP intoxication	ALT, AST, TP, Factor V	42/42	UPLC-QTOF/MS	Non-targeted + Targeted	Urine and plasma	Thiomethyl metabolites (S-CH3-APAP-S and SO-CH3-APAP-S)	None
Angela L. Chiew (2023) [16]	Australia	APAP	ALT > 1000 U/L	ALT	25/174	LC-MS/MS	Targeted	Serum	APAP-Cys and APAP-Mer.	APAP-Sul
Brandon J. Sonn (2022) [17]	USA	APAP	ALT elevation	ALT, ALKP, INR, TP, ALB, TB, DB, AST, TRIGER	(179+25)/ n*	UPLC-MS/MS	Non-targeted	Serum	Glycine, 5-oxoproline, 5-L-glutamyl-L-glutamine, L-citrulline, Ornithine, Allantoate and Nicotinamide.	None

James W Dear (2021) [18]	UK	NAC protection to APAP-overdose	APAP-ALT	ALT, INR, BILI, ALKP, GGT, UREA, CREA, Hb, MCV, WBC	(26 +19) /45	LC-MS/MS	Targeted	Plasma	ALI patients *vs* Non-ALI patients: APAP- cysteine, the ratio of APAP-cysteine/APAP (%), xanthine and its ratio (xanthine/ total purine metabolites [%])	None
Laura K. Schnackenberg (2017) [19]	USA	APAP	APAP-induced hepatotoxicity	ALT, APAP-protein Adduct	13/6	UPLC-QTOF/MS and NMR	Non-targeted	Urine	Overdose *vs* control: Hippurate, Ascorbic acid and Propylene glycol.	None
Nicola Gray (2017) [20]	UK	APAP	APAP-ALF	Not mentioned	14/40	UPLC-MS/MS	Targeted	Plasma and serum	APAP-ALF *vs* control: Sarcosine, Glutamine, Glycine, Phenylalanine and Tyrosine.	APAP-ALF *vs* control: Isoleucine and Valine.
Sudeepa Bhattacharyya (2016) [21]	USA	APAP	Hospitalized for APAP-overdose	ALT, APAP-protein Adduct	(16+8)/19	LC-MS/MS	Targeted	Serum	APAP-overdose *vs* control: LysoPC a C14:0 and Lyso PC a C16:1	APAP-overdose *vs* control: LysoPC a C24:0, LysoPC a C26:1, LysoPC a C28:1, PC aa C40.1, PC aa C40.2, PC aa C40.3, PC aa

										C42.6, PC ae C34.2, PC ae C34.3, PC ae C36.2, PC ae C36.3, PC ae C36.5, PC ae C38.2, PC ae C38.6, PC ae C42.0, PC ae C42.2 and PC ae C44.3.
Laura James (2015) [22]	USA	APAP	APAP-DILI	ALT, APAP-protein Adduct	(64+15) /19	LC-MS/MS	Targeted	Serum	APAP-overdose *vs* control: Taurodeoxycholic acid, Glycodeoxycholic acid and Glycoch enodeoxycholic acid	APAP-overdose *vs* control: Taurocholic acid and Cholic acid
Sudeepa Bhattacharyya (2014) [23]	USA	APAP	APAP-Hepatotoxi city	ALT, INR	(187+ 62) /23	UPLC-MS/MS	Targeted	Serum	APAP-overdose *vs* control: APAP protein adducts, Acetylcarnitine, Oleoylcarnitines and Palmitoylcarnitines	None
Mitchell R. McGill (2014)	USA	APAP	APAP-Liver injury	ALT, AST, PT, TB	30/6	UPLC-QTOF/M S	Targeted	Serum	Acylcarnitines	None

[24]										
Benjamin L. Woolbright (2014) [25]	USA	APAP	APAP-ALF	ALT, PT, ALP, TB	84/6	UPLC-QTOF/MS	Targeted	Serum and plasma	APAP-ALF *vs* control: Taurocholic acid, Glycocholic acid, Glycochenodeoxycholic acid, Taurochenodeoxycholic acid, Taurodeoxycholic acid, and Glycodeoxycholic acid; Non-surviving *vs* surviving: Glycodeoxycholic acid.	None
Ji Won Kim (2013) [26]	Korea	APAP	APAP-DILI	AST, ALT, ALP, TB, γ-GTP, LDH	20/20	NMR	Non-targeted	Urine	3-chlorotyrosine, Phenylalanine, and Glutarate;	Citrate, Glycine and Hippurate;
								plasma	Lactate, Glucose, 3-hydroxyisovalerate, Isoleucine, Acetylglycine, Acetone, Acetate, Glutamine, Ethanol and Isobutyrate	

Marlon J.A. Jetten (2012) [27]	The Netherlands	APAP	Abnormal liver function	ALT, TB, ALP, GGT, LDH, ALB	5/5	NMR, LC–MS and GC–MS	Non-targeted + Targeted	Serum and urine	High APAP-dose *vs* low APAP-dose: relatively more cysteine-APAP, hydroxy-APAP and methoxy-APAP.	None
JH Winnike (2010) [28]	USA	APAP	APAP induced hepatotoxicity	ALT, AST, α-GST	58/13	NMR	Non-targeted	Urine	Responders *vs* Nonresponders: Alanine, APAP, APAP-Cys, APAP-cysteine, Glycine, Met-His, Methyl-histidine, Acetate, APAP-Merc and APAP-mercapturate.	Responders *vs* Nonresponders: Creatine, Creatinine, Histidine, Betaine, Trimethylamine oxide and Hippurate.
Rick D. Fannin (2010) [29]	USA	APAP	APAP induced the metabolomic changes	ALT	6/3	NMR	Targeted	Serum	Lactate	None
T. Andrew Clayton (2009) [30]	UK	APAP	APAP induced the urinary metabolomic changes	Not mentioned	99/99	NMR	Non-targeted + Targeted	Urine	Individuals having high predose urinary levels of p-cresol sulfate had low postdose urinary ratios of APAP sulfate to APAP glucuronide.	None

n*, the sample size is not mentioned; None#, no specific differentially abundant metabolites were detected or mentioned; APAP, Acetaminophen; ALF, Acute liver failure; ALT, Alanine Aminotransferase; AST, Aspartate Aminotransferase; TP, Total Protein; ALKP, Alkaline Phosphatase; INR, International Normalized Ratio; ALB, Albumin; TB, Total Bilirubin; DB, Direct Bilirubin; BILI, Bilirubin; GGT, Gamma-Glutamyl Transferase; CREA, Creatinine; Hb, Hemoglobin; MCV, Mean Corpuscular Volume; WBC, White Blood Cell Count; PT, Prothrombin Time; γ-GTP, Gamma-Glutamyl Transpeptidase; LDH, Lactate Dehydrogenase; α-GST, Alpha Glutathione S-Transferase.

Supplementary Table S2: Characteristics of Chinese herbal medicine-related liver injury studies (*n* = 5)

First Author (Year) (Refs.)	Country	Medicine	Outcome	Biochemical test indicators	Case/ Control	Sample type	Analytic Platform	Targeted/ Non-targeted	Up-regulated biomarkers	Down-regulated biomarkers
Aizhen Xiong (2024) [31]	China	Pyrrolizidine alkaloids from herbal product	PAs-HSOS	ALT, AST, TB, DB, ALB, PT, D-Dimer, WBC, PL	60/30	Serum	UPLC-QTOF/ MS	Non-targeted + Targeted	Non-targeted: 5-L-Glutamyl-L-alanine, Phenylalanylphenylalanine, Glycochenodeoxycholate-3-sulfate or isomer, LysoPC(22:5) adduction, Cholestane-3,7,12,25-tetrol-3-glucuronide adduction, Taurocholic acid, Taurohyodeoxycholic acid, Glycochenodeoxycholate-3-sulfate, Glisoflavanone, Glycocholic acid, Glycoursodeoxycholic acid, PG (21:0/22:4) or isomer, Cervonoylethanolamide adduction,	Non-targeted: gamma-Glutamyl-beta-cyanoalanine, N-Acetylaminooctanoic acid, D-Myo-inositol 3,4,5,6-tetrakisphosphate, Myristic acid adduction, 2,4-diaminobutyric acid, C16 Sphinganine, Phytosphingosine, Dihydroxyeicosanoic acid adduction, LysoPC(20:4), LysoPC(18:2) or isomer, LysoPC(16:0), Sphinganine, LysoPC(0:0/18:0), LysoPC(18:0), Echinasteroside C 15-O-sulfate, Phytanic acid (FFA20:0) adduction, Palmitic acid (FFA16:0), Cholest-5-ene-3-beta,7-alp

	L-a-glutamyl-L-Lysine, Glycochenodeoxycholic acid, Glycodeoxycholic acid, Cervonoylethanolamide isomer, LPA(P-16:0e/0:0) adduction, Bilirubin, Hydroxy-hexadecanoic acid adduction, Cervonoylethanolamide isomer, 3-alpha-hydroxy-7-oxo-5 beta-cholanic acid adduction, LPA(P-16:0e/0:0) adduction, Glycoursodeoxycholic acid adduction, 9-hydroperoxy-10E,12-o ctadecadienoic acid, Linolenic acid (FFA18:1), Docosapentaenoic acid (FFA22:5), Angiotensin IV, Bilirubin Ixa,	ha-diol,I-Urobilin adduction, D-Urobilinogen adduction, Stearic acid (FFA18:0).

									PC(22:4/14:0) or isomer.<br>Targeted:<br>Cholic acid, Taurocholic acid, Glycocholic acid.	
Yinghuan Hu (2023) [32]	China	PM	PM-DILI	ALT, AST, TB, DB, GGT, ALP, TBA	6/13	Plasma	UPLC-MS/MS	Non-targeted	Hypoxanthine, Phenyl-anylphenylalanine, Deoxycholic acid, Taurochenodesoxycholic acid.	LysoPC(0:0/16:0), LysoPC(18:1(9Z)/0:0), Choline, D-Glutamic acid.
Ying Huang (2020) [33]	China	PM	PM-DILI	ALT, AST, ALP, TB, IgG, IgM	13/(12+24+9)	Plasma	UPLC-QTOF/MS	Non-targeted	DILI *vs* Control: valine, P-cresol, P-cresol sulfate, Oxalosuccinic acid, Homocysteine, L-1-lysophosphoethanolamine, Inosine.	Phosphatidylglycerol, Glycochenodeoxycholic acid, Lysophosphatidic acid.
Le Zhang (2020) [34]	China	PM	PM-DILI	ALT, AST, ALP, TB	6/30	Serum	UPLC-QTOF /MS	Non-targeted	Glyceric acid, l-glutamate, Oxalosuccinic acid, PC 22:6, Acylglyc-erone phosphate, Indole-5,6-quinone, Gentisate aldehyde, Precorrin 6Y, 2E-tetrade-cenoyl-CoA, Ceramide, 3-Indolyllactic acid,	Phosphoribo-syl-ATP.

									Crotonoyl-CoA, LysoPC 20:4, Stearic acid, PE 22:6, Precorrin 8X, Kynurenic acid, 4-Hydroxyphenylacetate, Glutaric acid, Phenyllactic acid, l-Tyrosine, l-Histidinol, Sphingomyelin, 5-l-Glutamyl-l-alanine.	
Jiabo Wang (2015) [35]	China	PM	PM-DILI	ALT, AST, GGT, ALP, LDH, TB, DB	20/(12+12)	Plasma	LC-QTOF/MS	Non-targeted	The high relativity metabolomic biomarkers of PM-DILI: LysoPC(18:2(9Z,12Z)), Farnesyl pyrophosphate, Oleoyl glycine, *trans*-Dodec-2-enoic acid, 20-Hydroxy-PGF2a, LysoPC(16:1(9Z)), PC(24:0/22:0).	None#

None#, no specific differentially abundant metabolites were detected or mentioned; PAs-HSOS, Pyrrolizidine alkaloids induced-Hepatic sinusoidal obstruction syndrome; PM-DILI, Polygonum multiflorum-induced liver injury; PL, platelets; TBA, Total bile acid; IgG, Immunoglobulin G; IgM, Immunoglobulin M.

Supplementary Table S3: Characteristics of exogenous chemical toxin-related liver injury studies (*n* = 3)

First Author (Year) (Refs.)	Country	Medicine	Outcome	Biochemical test indicators	Case/ Control	Sample type	Analytic Platform	Targeted/ Non-targeted	Up-regulated biomarkers	Down-regulated biomarkers
Meng Tian (2022) [36]	China	Cadmium	Cd-induced liver injury	ALT, AST, ALP, GGT, CHE, TB, DB, BILI, TBA	268/135	Serum	UPLC-MS/MS	Targeted	Glycoursodeoxycholic acid, Glycolithocholic acid, Taurolithocholic acid, Taurodeoxycholic acid.	None#
John J. Guardiola (2016) [37]	USA	Polyvinyl chloride	Highly exposed PVC	ALB, TB, ALP, AST, ALT, CK18 M30, CK18 M65.	17/27	Plasma	LC-MS/MS and GC/MS	Non-targeted	189 metabolites increased in the PVC exposure group: long chain (including arachidonic acid), essential (including linoleic acid) fatty acids, monohydroxy fatty acids (including 13-HODE), fatty acid dicarboxylates and oxidized arachidonic acid products (including 5, 9, and 15-HETE).	94 metabolites decreased in the PVC exposure group: carnitine and carnitine esters.

Huihui Wang (2011) [38]	China	Arsenic trioxide	Hepatotoxicity Induced by arsenic trioxide	ALT, AST, TP, ALB, WBC, RBC, PLT, HGB, HCT	23/23	Urine	Atomic absorption spectrophotometer	None	Arsenic trioxide treatment *vs* untreatment on day 10 *vs* day 0: inorganic arsenic, monomethylated arsenic, dimethylated arsenic, the total arsenic.	None

None#, no specific differentially abundant metabolites were detected or mentioned; Cd, Cadmium; PVC, Polyvinyl chloride. CHE, Cholinesterase; CK18 M30, Cytokeratin 18 M30 Fragment; CK18 M65, Cytokeratin 18 M65 Fragment; RBC, Red Blood Cell Count; PLT, Platelet Count; HGB, Hemoglobin; HCT, Hematocrit.

Supplementary Table S4: Characteristics of single chemical or class of chemicals-related liver injury studies (*n* = 11)

First Author (Year) (Refs.)	Country	Medicine	Outcome	Biochemical test indicators	Case / Control	Sample type	Analytic Platform	Targeted/ Non-targeted	Up-regulated biomarkers	Down-regulated biomarkers
Yi-Hsuan Chiang (2024) [39]	China (Taiwan)	Voriconazole	DILI	ALT, ALP, AST, GGT, TB	18/153	Plasma	LC-MS/MS	Targeted	Glycocholate and α-ketoglutarate.	None#
Ming-Gui Wang (2022) [40]	China	Anti-tuberculosis drug	ATB-DILI	AST, ALP, TB, ALB, CREA, UA, TG, Chol, HDLI, LDLI	30/30	Plasma	UPLC-MS/MS	Non-targeted	Nicotinic acid, Glycyl-l-leucine, 1-phenylethanol, Trigonelline, Hesperetin, Polygodial, 18-β-glycyrrhetinic acid, L-glutamic acid, Nicotinuric acid, 2-methylhippuric acid, Genistein, Glycochenodeoxycholate, PMe (52:1), PS (38:4), PS (36:1), PC (24:1/18:2).	Salicylic acid, Indole-3-pyruvic acid, Indole-3-lactic acid, Indole-3-acetaldehyde, 4′-methoxyacetophenone, Artemisinin, N4-acetylcytidine, Phenol, Propylparaben, Caprylic acid, Decanoic acid, TG (29:0/18:2/18:2), TG (29:0/18:1/18:2), PC (37:4) (rep)(rep).

Ming-Gui Wang (2022) [41]	China	Anti-tuberculosis drug	ATB-DILI	ALT, AST, TB, ALP, GT, ALB, CREA, UA, HGB, WBC, PLT	14/30	Urine	UPLC-MS/MS	Non-targeted	Choline,Trigonelline, N-acetylputrescine, N8-acetylspermidine, Glycocholate, Uric acid, Ecgonine, 1-methylnicotinamide, Sebacic acid, 3-hydroxyanthranilic acid, Mannitol, Lipoamide, Epinephrine, 11-dehydrothromboxane b2, Taurolithocholic acid 3-sulfate.	Pseudoephedrine, 6-methylquinoline, Picolinic acid, Carbendazim, Ophthalmic acid, Valerophenone, D-(-)-lyxose, Creatine, L-glutamic acid, Methylmalonic acid, Porphobilinogen, Heptanoic acid, Nonanoic acid.
Shouquan Wu (2022) [42]	China	Anti-tuberculosis drug	ATDH	ALT, TB, DB, ALP, ALB, LDH	33/41	Urine	GC-TOF-MS	Non-targeted	Creatine, Lactulose, Melibiose, Diglycerol and 4-hydroxy-3-methoxybenzoic acid;	Aminomalonic acid, Nicotinic acid, Lactobionic acid, D-glucoheptose, Ornithine and N-acetyl-L-glutamic acid.
Robert N Helsley (2022) [43]	USA	Alcohol	ALD	Not mentioned	285/5	Plasma	LC-MS/MS	Targeted	Trimethylamine, Betaine,γ-butyrobetaine and Carnitine	Trime-thylamine N-oxide

Xuesong Li (2020) [44]	China	Methimazole	Hepatotoxicity	ALT, AST, ALP, TB, DB, TR-Ab, TG-Ab, TPO-Ab, T3, T4, FT3, FT4	40/80	Plasma	GC–MS/MS	Targeted	Octadecanol, Octanoic acid, Lauric acid, Palmitic acid, Decanoic acid, Margaric acid, Glycerol, Ethanolamine, Octopamine, Putrescine, 2-Ketobutyric acid, Maleic acid, Glyceraldehyde.	Mannose, Cysteine, Serine, Ornithine, Methionine, Aconitic acid, Threonine, 2-Aminopimelic acid, Citric acid, 4-Hydroxyproline, Isoleucine, Hypotaurine, Asparagine, Leucine, Inositol, Creatinine, Inositol phosphate, Valine, Lysine, Thymine, Glycine, Erythritol, Arabitol, Phenylalanine, Proline, Threitol, Xylitol, Glucuronic acid, Uric acid.
Shin-Lun Wu (2020) [45]	China(Taiwan)	Voriconazole	Hepatotoxicity	ALT, AST, ALP, r-GTP, TB	12/53	Plasma	LC–MS/MS	Targeted	Cyti-dine, Inosine, Glycocholate, α-ketoglutarate, Pyruvate, and β-N-acet-ylglucosamine.	Tryptophan.

Jun Cao (2018) [46]	China	Anti-tuberculosis drugs (rifampicin, isoniazid, pyrazinamide, ethambutol)	DILI	ALT, AST, TB, UA	(49+11)/25	Urine	UPLC-MS/MS	Non-targeted	DILI group *vs* non-DILI group: Uric acid, 3-Furoic acid, 7-Cyano-7-deazaguanine, Sebacic acid, I-Methyl-4-nitro-imidazole, Mesaconic acid, cis-4-octenedioic acid, Imetit, D-Mannitol.	DILI group *vs* non-DILI group: Hypoxanthine, 3-hydroxy-3-methyl-Glutaric acid, Isobutyrylglycine, Pyrazineethanethiol, 2-Hydroxypropylphosphonate, p-Hydroxyphenylacetic acid, Gluconolactone, Indoxylsulfuric acid, Citramalic acid, Creatinine, L-Erythrulose, Orotinichalcone, Queuosine, N-acetylaspartate, cis-Aconitic acid, Urea, Myrigalone E, Glabrone, alpha-hydroxyisovaleric acid.

J Lee (2017) [47]	Korea	Amoxicillin/Clavulanate	AC-DILI	ALT, AST, ALP, TB, miR-122	31/31	Urine	UPLC-QTOF/MS	Non-targeted	Responder group *vs* Nonresponder group on day 8: 7-methylxanthine , 7-methyluric acid, 3-methylxanthine	Responder group *vs* Nonresponder group on day 8: Azelaic acid.
Taoguang Huo (2014) [48]	China	Valproate sodium	Hepatotoxicity	ALT, AST, GGT , ALP, TB, PA	Total 34	Serum	^{1}HNMR and UPLC–MS	Non-targeted	ANLFT group *vs* NLFT group: Uric acid, Leucine, Lactate, Alanine, Acetate, NAC, Acetoacetate, Glutamate, Pyruvate, Choline, Phenylalanine.	ANLFT group *vs* NLFT group: Creatine, LPC18:0, LPC 16:0, VLDL/LDL, LPC18:2, LPC18:1, LDL/VLDL, Citric acid, PC 16:0/22:6, PC 18:2/18:2, PC 16:0/18:2.
Ulf Andersson (2009) [49]	Sweden	Ximelagatran	ALT elevation	ALT	46/94	Plasma	NMR, HPLC-QTOF/MS and GC-MS	Non-targeted	Predisposition biomarkers: Formate, LCAT, Glutamic acid, APOA2, APOE; Treatment-effect biomarkers: 3-Hydroxy butyrate, CSF1R, PROS1, L-Phenylalanine,	Predisposition biomarkers: KNG1, L-Cystine, Pyruvic acid, Alanine, 2-Ketoglutaric acid, APOA4; Treatment-effect biomarkers: Pyruvic acid,

	C4BPA, Tyrosine.	L-glutamine, Vitamin E, Gc-globulin.

None#, no specific differentially abundant metabolites were detected or mentioned; ATB, Anti-tuberculosis drug; ATDH, Anti-tuberculosis drug-induced hepatotoxicity; ALD, Alcohol- associated liver diseases. UA, Uric Acid; TG, Triglyceride; Chol, Cholesterol; HDLI, High-Density Lipoprotein; LDLI, Low-Density Lipoprotein; LDH, Lactate Dehydrogenase; TR-Ab, Thyroid Stimulating Hormone Receptor Antibody; TG-Ab, Thyroglobulin Antibodies; TPO-Ab, Thyroid Peroxidase Antibodies; T3, Triiodothyronine; T4, Thyroxine; FT3, Free Triiodothyronine; FT4, Free Thyroxine; PA, Prealbumin.

Supplementary Table S5: Characteristics of multidrug-related liver injury studies (*n* = 19)

First Author (Year) (Refs.)	Country	Medicine	Outcome	Biochemical test indicators	Case / Control	Sample type	Analytic Platform	Targeted/ Non-targeted	Up-regulated biomarkers	Down-regulated biomarkers
Marta Moreno-Torres (2024) [50]	Spain	Epistance, Oxaliplatin, Azathioprine, Acetaminophen, Amox-Clav, Methotrexate	DILI	ALT, ALP, TB, GGT, ALB	167/111	Plasma	UPLC-QTOF/MS	Non-targeted	Metabolite prediction models characterized the distinct DILI phenotypes: free and conjugated bile acids and glycerophospholipids.	None[#]
Kaini He (2023) [51]	China	Not mentioned	DILI	Not mentioned	28/28	Faeces	PacBio Sequencing	None	Metabolites: 10 up-regulated metabolites	Metabolites: 56 down-regulated metabolites
Francisco Andújar-Vera (2023) [52]	Spain	Horsetail (Equisetum), Green anise, Amoxicillin-clavulanic acid, Methotrexate, Ibuprofen, Isoniacid, Acyclovir, Cotrimoxazole, Cloxacillin, Amoxicillin,	DILI	ALT, ALP	32/66	Plasma	LC-QTOF/MS	Non-targeted	Lysophosphatidylcholine (16:1/0:0), Glycocholic acid, Taurocholic acid.	Hydroxydecanoylcarnitine, Octanoylcarnitine.

		Voriconazole, Zonisamide, Meropenem, Propranolol, Amiodarone, Rifampicin							
Shuang Zhao (2022) [53]	China	Conventional drugs and herbs	DILI	ALT, AST, ALP, GGT, TB, DB, INR, AFP, TBA	90/70	Serum	UPLC-MS/MS	Targeted	DILI *vs* control: Glycochenodeoxycholic acid, Glycocholic acid, Taurochenodeoxycholic acid, Taurocholic acid; Severe DILI *vs* Slight DILI: BAs, short chain fatty acids (SCFAs), fatty acids; DILI.chr *vs* DILI.rec: a-Linolenic acid, Glinolenic acid , Linoleic acid, Adrenic acid, Docosapentaenoic acid, DPAn-6.	None

Xian He (2022) [54]	China	Not mentioned	Chronic DILI	ALT, AST, ALP, GGT, TB, DB, ALB, CHE, TC, TG, INR, CREA, PT	96/11	Serum	LC-QTOF/MS	Non-targeted	Metabolite fingerprint associated with DILI fibrosis: Cortolone-3-glucuronide, 5-Cholesten-3beta-25-diol-3-sulfate, Tetrahydroaldosterone-3-glucuronide, Cer(d18:0/23:0), Citric acid, N-acetyl-seryl-aspartate, PG(PGE1/18:1(9Z)), PA(18:3(9,11,15)-OH(13)/i-13:0), 3(S)-Hydroxy-docosa-10,13,16,19-all-cis-tetraenoyl-CoA, TG(18:2(9Z,12Z)/20:0/20:0), PGP(a-25:0/PGF2alpha), PIP(20:4(8Z,11Z,14Z,17Z)/5-iso PGF2VI), PG(i-12:0/LTE4), Ganoderic acid L,	Metabolite fingerprint associated with DILI fibrosis: Lithocholic acid glycine conjugate, O-methoxycatechol-O sulphate, Isodeoxycholic acid, PA(10:0/20:4(6E,8Z,11Z,14Z)+=O(5)), PC(20:3(5Z,8Z,11Z)/20:4(8Z,11Z,14Z,17Z)), PI(22:2(13Z,16Z)/22:6(5Z,7Z,10Z,13Z,16Z,19Z)-OH(4)), LysoPC(14:0), N-Myristoyl Tryptophan, Glycochenodeoxycholic acid 3-glucuronide, N-Eicosapentaenoyl Isoleucine, Leukotriene C4, PC(2:0/PGD2), PE(22:0/LTE4), LysoSM(d18:0),

									Furocarpic acid ; Metabolite fingerprint associated with DILI advanced-fibrosis: TG(20:0/24:1(15Z)/18:1(9Z)), TG(14:0/24:0/24:1(15Z)), PI(18:0/16:0), PA(a-21:0/i-18:0), 3-Hexaprenyl-4-hydroxy-5-methoxybenzoic acid, TG(22:0/i-24:0/i-24:0), TG(22:0/18:2(9Z,12Z)/16:1(9Z)), PE(P-16:0/14:0).	Glycerylmonooleate, Tetradecanedioic acid; Metabolite fingerprint associated with DILI advanced-fibrosis: Homovanillic acid sulfate, PE-NMe(20:0/22:6(4Z,7Z,10Z,13Z,16Z,19Z)), TG(24:0/20:4(5Z,8Z,11Z,14Z)/22:1(13Z)).
Yan-zhong Han (2022) [55]	China	DILI patients	AIH, DILI^{Ab+}, DILI^{Ab-}	ALT, AST, ALP, GGT, TB, DB, IgA, IgG, IgM, CHE, INR, TC, TG, ALB, WBC, PLT, TBA, Cr, PT, γ-Globulin	(59+68+75)/n*	Serum	LC-QTOF/MS	Non-targeted	AIH *vs* DILI^{Ab-}: 388 differential metabolites, 74 fingerprint metabolites.	None

Xinbei Tian (2022) [56]	China	Biliary atresia patients	Biliary atresia	ALT, AST, GGT, TB, DB, TBA	46/22	Plasma	LC-MS/MS	Targeted	D-2-hydroxyglutarate.	None
Zhongyang Xie (2021) [57]	China	Polygonum Multiflorum, Fructus Psoraleae, Anti-Tuberculosis, Cephalosporin, Azithromycin, Amoxicillin	DILI patients	ALT, AST, ALP, GGT, TBA, ALB, TB, TG, HDL, LDL, VLDL, WBC, RBC, PLT, Chol, INR	161/31	Serum	UPLC-MS/MS	Targeted	DILI group *vs* control group: Glycocholic acid, Glycochenodeoxycholic acid, Nor- Cholic acid, Taurocholic acid, Taurochenodeoxycholic acid.	DILI group *vs* control group: 12-keto Lithocholic acid, b Ursodeoxycholic acid, Deoxycholic acid.
Shuai-shuai Chen (2021) [58]	China	Not mentioned	Chronic DILI with or without cirrhosis	ALT, AST, ALP, TB, DB, TBA, INR, γ-GTP, CHE, TC, TG, Cr, IgA, IgG, IgM, WBC, PLT, ALB, GLOB, ALB/GLOB, PA	83/10	Serum	LC-QTOF/MS	Non-targeted	Cirrhosis-Related Metabolic Fingerprint from HS, NC, and CC to DC have been on the rise: 5alpha-Androstan-3beta, 17alpha-diol disulfate, 2-(3-Carboxy-3-aopropyl)-L-histidine, 3-Methoxyanthranilate, Taurochenodesoxycholic acid, L-Phenylalanine,	None

Tuftsin, D-Xylulose, Citric acid, Beta-Citryl-L-glutamic acid, 9-Hydroxypalmitic acid, 3-keto stearic acid, Glycocholic acid, PC(22:6(4Z,7Z,10Z,13Z,16Z,19Z)/18:3(9Z,12Z,15Z)), Chenodeoxycholic acid Glycine conjugate, Propionic acid, SM(d18:0/16:1(9Z)(OH)), PC(14:0/20:0), 3-Hydroxyanthranilic acid, (all-Z)-8,11,14-Heptadecatrienal, N(omega)-Hydroxyarginine, 5-Methyltetrahydrofolic acid, N1-Acetylspermidine, Indolepyruvate, L-Arginine,

									Dihydrouracil, Tryptophyl-Glutae, Cysteinyl-Proline, Enol-phenylpyruvate, Indoleacetaldehyde, 7-Methylguanine.	
Guillermo Quintás (2021) [59]	Spain	Epistane, Levofloxacin, Amoxi-Clav, Atorvastatin, Rosuvastatina, Metildopa, Levofloxacin, Acetaminophen, IFN beta1a, Herbs, Cefuroxime, Azathioprine, Adalimumab, Ciprofloxacin, Nitrofurantoin, Voriconazole, Topiramate, Fenofibrate, Teriflunomide, Ceftriax,	DILI	ALT, AST, ALP, GGT, TB, ALB, TG, Chol, Glu, Cr	168/115	Serum	UPLC-MS/MS	Non-targeted	The mixed DILI group *vs* recovered group: Glycerophosphoetha-n olamines, Glycerophosphocholine s; The cholestasic DILI group *vs* recovered group: Bile acids and Derivatives; The cholestasic DILI group *vs* other group: Bile acids, Amino acids, Glycerophosphocholine s, Steroidal glycosides.	None

		Methotrexate, Temozolomide, Trabectedin, Ibuprofen, Oxaliplatin, Drugs-illegal, Vitamin A, Dexketoprofen, Isoniazid, Trabectedin							
Kosuke Saito (2020) [60]	Japan	Loxoprofen, Antibacterial agents, Antineoplastic agents, Antiinflammatory and Antirheumatic products, Psycholeptics	DILI	ALT, ALP, AST, TB	54/60	Plasma	RPLC-MS/MS	Non-targeted	The mixed-type DILI group *vs* healthy subjects: Cer(d34:1; d18:1/16:0), Cer(d36:1; d18:1/18:0), GM3(d34:1)+O; The cholestatic-type DILI *vs* healthy subjects: PC(30:0;14:0/16:0), PC(31:0;15:0/16:0), PC(32:1; 16:0/16:1), PC(33:1;15:0/18:1,16:0/17:1).	The mixed-type DILI group *vs* healthy subjects: LPC(18:2), LPC(16:1e), PE(38:6e;18:2e/20:4). The cholestatic-type DILI *vs* healthy subjects: SM(d40:1), PI(38:4), PI(38:3) and PC(36:5e).

Zhenhua Ma (2019) [61]	China	Traditional Chinese medicine, Analgesic-antipyretic drugs, Antituberculosis drugs, Antibiotic drug, Weight-reducing medicine, Antihypertensive drugs, Lipid-lowering drugs, Tibetan medicine, Healthcare products.	DILI	ALT, AST, ALP, g-GT , TB, DB, TBA, INR	38/30	Serum	UPLC-MS/MS	Non-targeted +Targeted	DILI *vs* control: Taurocholic acid, Taurochenodeoxycholic acids and Palmitic amide;<br>Severe DILI *vs* control: Glycocholic acid, Taurocholic acid, Tauroursodeoxycholic acid, Glycochenodeoxycholic acid, Glycochenodeoxycholic sulfate, Taurodeoxycholic acid.	DILI *vs* control: LysoPE (18:0/0:0).<br>Severe DILI *vs* control: Chenodeoxycholic acid, Deoxycholic acid, Lithocholic acid.

Zhongyang Xie (2019) [62]	China	Clarithromycin, Cephalosporin, Amoxicillin, Piperacillin-tazobactam, Capecitabine, Sodium valproate, Ibuprofen, TNF-αantagonist, Isoniazid/Rifampicin/Pyrazinamide, Acyclovir, Rabeprazole, Rabies vaccines, Phenobarbital.	DILI	ALP, ALT, AST, INR, TB, keratin-18	58/34	Serum	GC−MS and UPLC−MS	Non-targeted	Severe *vs* non-severe groups: Glycochenodeoxycholic acid, Taurochenodeoxycholic acid, Sphingomyelin.	Severe *vs* non-severe groups: Cholesterol, Linolenic acid, Glycerol 3-phosphate, Phosphatidylcholine, PDGF-bb, IP-10, IL-1Rα, MIP-1β, TNF-α.
Xiaohui Wang (2019) [63]	China	Not mentioned	ALD, DILI, ALD-DILI	ALT, AST, ALP, TB, GGT, TC, TG, INR, ALB, CHE	(10+40)/40	Serum	HPLC-QTOF /MS	Non-targeted	DILI *vs* ALD-DILI : PC(16:1(9Z)/14:1(9Z)), PS(18:1(9Z)/18:0)[U], PG(22:1(11Z)/15:0), PA(21:0/22:6 (4Z,7Z,10Z,13Z,16Z,19Z)), Indole-3-acetate.	DILI *vs* ALD-DILI : Taurolithocholate, 3-Ketolactose, 5, 10-Methenyltetrahydrofolate, PE(18:1(11Z)/15:0), (R)-Lactate, Taurine, L-Tryptophan.

Luge Wei (2019) [64]	China	Not mentioned	LC-DILI and CH-DILI	ALT, AST, ALP, TB, DB, ChE, CREA, IgA, IgG, IgM, INR	15/34	Serum	HPLC-QTOF /MS	Non-targeted	Chenodeoxycholic acid glycine conjugate, Pectic acid, Taurochenodeoxycholic acid, 3-Dehydro-L-gulonate, LysoPC (18:1 (9Z)), Glycocholic acid, D-Glucuronic acid, Taurocholic acid, Protoporphyrin IX, Creatine, 2-Oxoarginine, 5b-Cyprinol sulfate, 2-Hydroxyethanesulfonate, D-Xylonate, L-Galactonate, 3-Methoxy-4-hydroxyphenylglycolaldehyde, Deoxyribose, 3-(4-Hydroxyphenyl) lactic acid, Aldosterone, 1,2-Dihydroxy-3-keto-5-methylthiopentene, Dihydrothymine,	Phosphatidylcholine, PE (15:0/22:1 (13Z)), PA (24:0/20:3 (8Z, 11Z, 14Z)).

									5-Hydroxyisourate, 5-L-Glutamyl-taurine, Homovanillin, Cholesterol sulfate, LysoPC (18:4 (6Z,9Z,12Z,15Z)), LysoPC (16:1 (9Z)/0:0), LysoPE (20:3 (5Z,8Z,11Z)/0:0), LysoPC (15:0), Menaquinol, PA (18:3 (9Z,12Z,15Z)/24:0), Indoleacetaldehyde.	
Zhuo-Ling An (2015) [65]	China	Antitumor drugs, Traditional Chinese medicine, Statins durg and Antimicrobials	DILI	AST, ALT	24/21	Serum	UPLC-MS/MS	Non-targeted	Dimethylguanosine, Phenylalanine.	None
Jiabo Wang (2014) [66]	China	Not mentioned	AIH, DILI	ALT, AST, ALP, IgG, IgM, ANA, ASMA, AMA	(39+14)/23	Plasma	^{1}HNMR	Non-targeted	AIH vs (healthy subjects and DILI): Acetoacetate, Acetone, β-hydroxyisobutyrate, Citrate, Creatine, Dimethylamine,	AIH *vs* healthy subjects: Alanine, Isoleucine, Leucine, Valine.

									Glutamine, Histidine, Pyruvate.	
Xiaofen Li (2013) [67]	China	Chinese medicines, antituberculosis drugs, antithyroid drugs, chemotherapy drugs and antipsychotic drugs	DILI	ALT, TB, PT	26/23	Serum	UPLC-QTOF/MS	Non-targeted	Stearamide, Oleamide, Tetradecanamide, Lecithin, Glycochenodeoxycholicacid ,Glycocholic acid, Bilirubin, Hypoxanthine.	LPC C16:0, LPC C18:0, LPC C18:3, LPC C18:2
Tomoyoshi Soga (2011) [68]	Japan	Not mentioned	DILI	AST, ALT, γ-GTP, AFP, PIVKA-II	27/53	Serum	CE-TOF/MS and LC-MS/MS	Non-targeted +Targeted	DILI group *vs* control group: γ-glutamyl dipeptides, Oxidative metabolites, Methionine sulfoxide, Glucosamine.	None

None#, no specific differentially abundant metabolites were detected or mentioned; n*, the sample size is not mentioned; AIH, Autoimmune hepatitis; DILI^{Ab+}, DILI with autoantibodies; DILI^{Ab-}, DILI without autoantibodies; ATB, Anti-tuberculosis drug; ATDH, Anti-tuberculosis drug-induced hepatotoxicity; ALD, Alcohol- associated liver diseases; CH-DILI, Chronic manifestation- drug-induced liver injury; LC-DILI, Liver cirrhosis- drug-induced liver injury; AFP, Alpha Fetoprotein; TC, Total Cholesterol; IgA, Immunoglobulin A; VLDL, Very Low-Density Lipoprotein; GLOB, Globulin; Glu, Glucose; ANA, Anti-Nuclear Antibody; ASMA, Anti-Smooth Muscle Antibody; AMA, Anti-Mitochondrial Antibody; PIVKA-II, Protein Induced by Vitamin K Absence or Antagonist-II.
